# Effect of inspiratory synchronization during pressure-controlled ventilation on lung distension and inspiratory effort

**DOI:** 10.1186/s13613-017-0324-z

**Published:** 2017-10-06

**Authors:** Nuttapol Rittayamai, François Beloncle, Ewan C. Goligher, Lu Chen, Jordi Mancebo, Jean-Christophe M. Richard, Laurent Brochard

**Affiliations:** 1 Interdepartmental Division of Critical Care Medicine, University of Toronto, Toronto, ON Canada; 2 Keenan Research Centre and Li Ka Shing Knowledge Institute, St. Michael’s Hospital, 30 Bond St, Toronto, ON M5B 1W8 Canada; 3 Division of Respiratory Diseases and Tuberculosis, Department of Medicine, Faculty of Medicine Siriraj Hospital, Bangkok, Thailand; 4 Medical Intensive Care Unit, Hospital of Angers, University of Angers, Angers, France; 5 Department of Medicine, University of Toronto, Toronto, Canada; 6 Department of Physiology, University of Toronto, Toronto, Canada; 7 Division of Respirology, Department of Medicine, University Health Network and Mount Sinai Hospital, Toronto, Canada; 8 Centre de recherche du Centre Hospitalier de l, Université de Montréal (CRCHUM), University of Montreal’, Montreal, Canada; 9 Servei de Medicina Intensiva, Hospital Sant Pau, Barcelona, Spain; 10 Emergency Department, General Hospital of Annecy, Annecy, France; 11 INSERM UMR 955 eq 13, Créteil, France

**Keywords:** Airway pressure release ventilation, Lung-protective ventilation, Spontaneous ventilation, Transpulmonary pressure, Ventilator-induced lung injury

## Abstract

**Background:**

In pressure-controlled (PC) ventilation, tidal volume (*V*_T_) and transpulmonary pressure (*P*_*L*_) result from the addition of ventilator pressure and the patient’s inspiratory effort. PC modes can be classified into fully, partially, and non-synchronized modes, and the degree of synchronization may result in different *V*_T_ and *P*_*L*_ despite identical ventilator settings. This study assessed the effects of three PC modes on *V*_T_, *P*_*L*_, inspiratory effort (esophageal pressure–time product, PTP_es_), and airway occlusion pressure, *P*_0.1_. We also assessed whether *P*_0.1_ can be used for evaluating patient effort.

**Methods:**

Prospective, randomized, crossover physiologic study performed in 14 spontaneously breathing mechanically ventilated patients recovering from acute respiratory failure (1 subsequently withdrew). PC modes were fully (PC-CMV), partially (PC-SIMV), and non-synchronized (PC-IMV using airway pressure release ventilation) and were applied randomly; driving pressure, inspiratory time, and set respiratory rate being similar for all modes. Airway, esophageal pressure, *P*_0.1_, airflow, gas exchange, and hemodynamics were recorded.

**Results:**

*V*_T_ was significantly lower during PC-IMV as compared with PC-SIMV and PC-CMV (387 ± 105 vs 458 ± 134 vs 482 ± 108 mL, respectively; *p* < 0.05). Maximal *P*_*L*_ was also significantly lower (13.3 ± 4.9 vs 15.3 ± 5.7 vs 15.5 ± 5.2 cmH_2_O, respectively; *p* < 0.05), but PTP_es_ was significantly higher in PC-IMV (215.6 ± 154.3 vs 150.0 ± 102.4 vs 130.9 ± 101.8 cmH_2_O × s × min^−1^, respectively; *p* < 0.05), with no differences in gas exchange and hemodynamic variables. PTP_es_ increased by more than 15% in 10 patients and by more than 50% in 5 patients. An increased *P*_0.1_ could identify high levels of PTP_es_.

**Conclusions:**

Non-synchronized PC mode lowers *V*_T_ and *P*_*L*_ in comparison with more synchronized modes in spontaneously breathing patients but can increase patient effort and may need specific adjustments.

*Clinical Trial Registration* Clinicaltrial.gov # NCT02071277

**Electronic supplementary material:**

The online version of this article (doi:10.1186/s13613-017-0324-z) contains supplementary material, which is available to authorized users.

## Background

To date, volume-controlled ventilation is the most commonly employed mode during the first few days of mechanical ventilation [1]. The use of pressure-controlled (PC) modes has steadily increased, and they are now preferentially used. Under passive conditions in PC mode, the ventilator is the only respiratory pump and *V*_T_ depends entirely on the set pressure, inspiratory time, and the respiratory system mechanics [2]. Inactivity of the respiratory muscles results in rapid muscle weakness [3, 4], whereas allowing spontaneous breathing improves gas exchange [5] and might prevent ventilator-induced diaphragm dysfunction (VIDD) [6, 7]. When patients make spontaneous breathing efforts, however, the total driving pressure will be the sum of the pressure generated by the ventilator (*P*_aw_) and the patient’s respiratory muscles. Therefore, transpulmonary pressure (*P*_*L*_) and *V*_T_ are more difficult to control and may exceed safe limits in patients who require lung-protective ventilation, such as acute respiratory distress syndrome (ARDS).

**Fig. 1:**
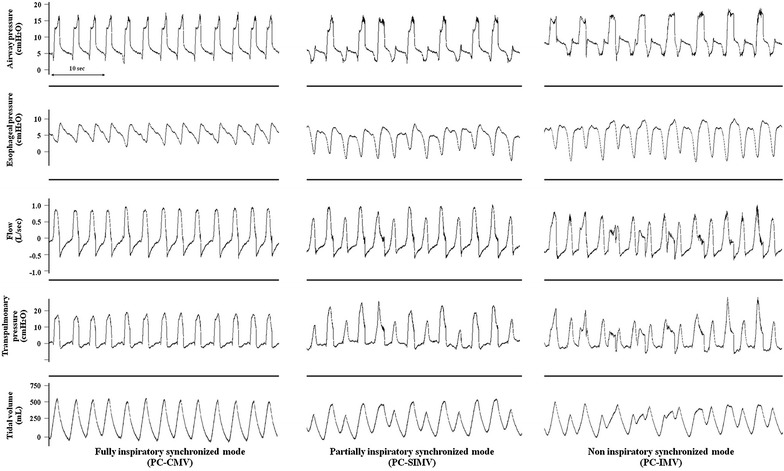
Tracings of airway pressure, esophageal pressure, flow, transpulmonary pressure, and tidal volume during each pressure-controlled mode of ventilation. The degree of inspiratory synchronization leads to varying in transpulmonary pressure and tidal volume. *PC-CMV* pressure-controlled continuous mandatory ventilation, *PC-SIMV* pressure-controlled synchronized intermittent mandatory ventilation, *PC-IMV* pressure-controlled intermittent mandatory ventilation

Pressure-controlled modes can be classified according to the degree of inspiratory synchronization as fully, partially, and non-synchronized modes (Fig. 1). The nomenclature of each mode, however, varies with ventilator brand making sometimes difficult for the clinician to appreciate this distinction (Additional file 1: Table S1). In fully synchronized mode or PC continuous mandatory ventilation (PC-CMV), mechanically assisted breaths are triggered every time the patient generates spontaneous efforts. In partially synchronized mode or PC synchronized intermittent mandatory ventilation (PC-SIMV), there is a synchronization time window allowing the patient to trigger an assisted breath within the time window or to take a breath without assistance if efforts occur outside the synchronization window. Finally, in non-synchronized mode or PC intermittent mandatory ventilation (PC-IMV), low and high pressure levels are alternately delivered for fixed intervals and patient inspiratory efforts are possible but do not trigger any additional assistance and are not intentionally synchronized. Several breath types can be observed during PC-IMV, which will result in different breathing patterns (Additional file 1: Fig. S1) [8]. A study by Richard and colleagues comparing three PC types of modes in a bench model suggested that non-synchronized modes resulted in lower *P*_*L*_ and *V*_T_ than the two other modes despite identical settings and simulated effort [9]. Though these effects are potentially attractive for offering a better lung-protective strategy, using a non-synchronized mode may also lead to unpredictable effects on patient’s inspiratory effort. Because we don’t know if the risk of having large *V*_T_ and *P*_*L*_ is better represented by the average values, the variability of the values needs to be also examined.

The pressure–time product (PTP) and work of breathing using Campbell’s diagram are the standard methods for assessing patient inspiratory effort during mechanical ventilation [10]. However, these techniques need complex calculations based on esophageal manometry. The airway occlusion pressure at 0.1 s (*P*_0.1_), an index of respiratory drive available on modern ventilators, could be an alternative method for assessing inspiratory effort.

The primary objective of this study was to assess whether non-synchronized modes of ventilation result in more protective ventilation strategy over the two other PC modes as evaluated by *V*_T_ and *P*_*L*_; secondary objectives included the effect of different degree of inspiratory synchronization on inspiratory effort determined by esophageal pressure–time product (PTP_es_) and by *P*_0.1_.

## Methods

### Study population and settings

The study was conducted in Medical–Surgical Intensive Care Units at two academic hospitals in Toronto, Canada (Clinicaltrial.gov # NCT02071277). The Research Ethics Board at St. Michael’s Hospital and Mt. Sinai Hospital approved the study protocol, and informed consent was obtained from patients or their substitute decision makers prior to enrollment.

Patients were eligible for enrollment if they were spontaneously breathing under mechanical ventilation with a pressure assist-control mode or pressure support ventilation (PSV) with a ventilator driving pressure level of at least 10 cmH_2_O (to ensure that patients were not yet on minimal support). Patients were not included if they had hemodynamic instability (> 20% variation of mean arterial pressure and/or heart rate or need doses of norepinephrine higher than 0.2 mcg/kg/min), a set positive end-expiratory pressure (PEEP) above 12 cmH_2_O, a fractional oxygen concentration (FiO_2_) above 0.6, a severe acid–base disturbance (arterial pH < 7.30 or > 7.55). There should be no contraindication to insert esophageal balloon catheter, chronic neuromuscular disease, intracranial hypertension, or pregnancy.

### Ventilators and equipment

A Dräger Evita-XL or a Dräger V500 ventilator (Dräger, Lubeck, Germany) which provided the three different synchronized PC modes was used. We used PCV+ assist, PCV+, and APRV modes on the Evita-XL and PC-AC, PC-SIMV+, and APRV on the V500 ventilator to represent PC-CMV, PC-SIMV, and PC-IMV, respectively. Of note, we used the mode called APRV as the only available non-synchronized mode, but the settings were similar to other classical PC-CMV modes and not to “usual” approaches using APRV with prolonged high pressure–time.

Airflow was measured with a Fleisch No. 2 pneumotachograph placed between the endotracheal tube and the Y-piece of the ventilator, connected to a differential pressure transducer (MP 150, Biopac Systems, Goleta, California, USA). Airway pressure (*P*_aw_) was measured between the endotracheal tube and the pneumotachograph via a pressure transducer (MP 150). Esophageal pressure (*P*_es_) was measured using a Nutrivent catheter (Sidam, Mirandola, Italy) connected to pressure transducers (MP 150). The correct position of the esophageal balloon was assessed by an occlusion test [11, 12].

The analog signals of airflow, *P*_aw_, and *P*_es_ were digitized at a sampling rate of 100 Hz and stored in a laptop for subsequent calculations and analyzes using AcqKnowledge software (Biopac Systems). Volume was obtained by integration of airflow signal over time, regardless of the mode. Tidal volume variability was assessed by the coefficients of variation of tidal volume (calculated as the standard deviation divided by the mean value). *P*_*L*_ was calculated by subtraction of *P*_aw_ from *P*_es_ and presented as the maximal and minimal values; mean *P*_*L*_ was calculated as the quotient of the area under the *P*_*L*_-time tracing divided by total cycle duration. Δ*P*_*L*_ was measured as the difference between maximal and minimal *P*_*L*_ at the end of inspiration. PTP_es_ was calculated as the surface enclosed within the *P*_es_ and the relaxation line of the chest wall over inspiratory time [13, 14] and expressed in cmH_2_O × s × min^−1^ using a dedicated software (Sistema Respiratorio, Barcelona, Spain). Algorithm to calculate PTP_es_ is detailed in Additional file 1: Fig. S2.

*P*_0.1_ was measured using AcqKnowledge software from the fall in *P*_aw_ during the first 100 ms of an occluded (zero flow) spontaneous inspiration using the end-expiratory hold function.

### Study protocol

Patients were studied in a semi-recumbent position. Three different PC modes were applied for 20 min each in random order as determined by a blind envelope pull. The ventilator settings (inspiratory pressure, PEEP, set respiratory rate, FiO_2_, and inspiratory time) were kept unchanged and similar across all modes. These settings were as close as possible to those previously chosen by the responsible clinician, using the same driving pressure; if the patient was put on PSV mode, then the set respiratory rate during the study was set to reach the same total minute ventilation. No pressure support was added during PC-SIMV and PC-IMV. The first 15 min was devoted to ensure patient’s full adaptation to the mode, and signal acquisition was done during the following 5 min. The last 2 min of the recording was analyzed offline and presented as the average values over the selected period. Sedation assessed by RASS was left to the discretion of the attended physician and not modified for the duration of the study. The occlusions to measure *P*_0.1_ were performed and recorded every minute during 5 min of data acquisition. Arterial blood gases were collected before starting the protocol and at the end of the three studied periods. Hemodynamic variables (mean arterial pressure and heart rate) were also recorded during the study.

### Statistical analysis

Statistical analysis was performed with Statistical Package for the Social Sciences (version 20.0, IBM SPSS, Chicago, IL, USA). Continuous variables are reported as mean ± SD, and categorical variables are reported as number and percentage. We used an analysis of variance with repeated measures followed by a post hoc pairwise test to compare the difference between the three modes.

We also performed a correlation analysis between the individual changes in *P*_0.1_ and the individual changes in PTP_es_ in order to determine whether *P*_0.1_ could reliably indicate the direction of the changes in patients’ effort. Receiver operating characteristic (ROC) curve was used to evaluate a cutoff point for *P*_0.1_ in predicting excess patient’s inspiratory effort determined as PTP_es_ > 200 cmH_2_O × s × min^−1^. This value was chosen as the upper value, i.e., mean value plus one standard deviation, tolerated by patients passing a successful spontaneous breathing trial [14]. A *p* value < 0.05 was considered as statistically significant.

## Results

We enrolled 14 patients from March 2014 to July 2015. Mean age was 58 ± 12 years, and APACHE II score was 18.0 ± 5.1. Other baseline characteristics are shown in Table 1. The majority of patients (62%) had been ventilated for ARDS, and 46% were still under light levels of continuous intravenous sedation at the time of the measurements. (Average RASS score of these patients was −2 ± 1.) All but one patient tolerated the three PC modes. The latter patient was in respiratory acidosis before the study, worsened just after starting the study, and was secondarily excluded.

**Table 1:** Patient characteristics and ventilator settings

Patient	Gender	Age (years)	Cause of acute respiratory failure	Intubation days	APACHE II score	RASS score	Inspiratory pressure above PEEP (cmH_2_O)	PEEP (cmH_2_O)	Inspiratory time (s)	Set rate (breath/min)	FiO_2_	Discharge status
1	M	62	Sepsis, ARDS	14	12	−2	10	8	1	15	0.5	Alive
2*	F	65	COPD with exacerbation	2	13	0	12	5	0.9	26	0.45	Alive
3	M	80	COPD with exacerbation	15	13	0	10	8	1.1	19	0.3	Alive
4	M	66	Sepsis, ARDS	3	16	−2	16	8	1	20	0.4	Alive
5	M	68	Congestive heart failure	4	16	−2	12	10	1	20	0.5	Alive
6	M	48	Pneumonia, ARDS	13	17	−2	10	10	0.9	14	0.4	Dead
7	M	38	Multiple trauma	7	11	−3	12	8	1	16	0.4	Alive
8	F	41	Seizure, ARDS	7	20	−3	10	8	1	18	0.4	Alive
9	M	69	Sepsis, ARDS	9	18	−3	18	10	0.8	25	0.5	Dead
10	M	46	IPF exacerbation, ARDS	10	19	−3	20	8	0.8	24	0.5	Alive
11	F	67	Cardiac arrest	7	19	−3	14	12	1	20	0.5	Alive
12	M	49	Sepsis, ARDS	8	24	−3	14	12	1	22	0.4	Alive
13	M	63	Pneumonia, ARDS	2	25	−1	16	10	0.9	13	0.4	Dead
14	F	51	Pneumonia	11	29	−3	16	10	1.2	14	0.45	Dead

*ARDS* acute respiratory distress syndrome, *COPD* chronic obstructive pulmonary disease, *IPF* idiopathic pulmonary fibrosis, *PEEP* positive end-expiratory pressure, *RASS* Richmond Agitation Sedation Scale

* Patient #2 was excluded from the data analysis due to termination of the study

### Effect on breathing pattern and transpulmonary pressure

**Fig. 2:**
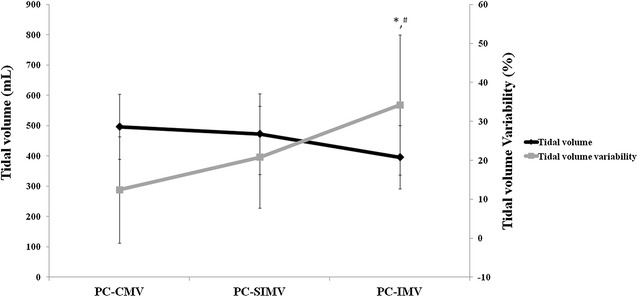
Tidal volume and tidal volume variability during fully, partially, and non inspiratory synchronized pressure-controlled modes (*p < 0.05; PC-IMV vs PC-CMV and ^#^ *p* < 0.05; PC-IMV vs PC-SIMV). *PC-CMV* pressure-controlled continuous mandatory ventilation, *PC-SIMV* pressure-controlled synchronized intermittent mandatory ventilation, *PC-IMV* pressure-controlled intermittent mandatory ventilation

**Fig. 3:**
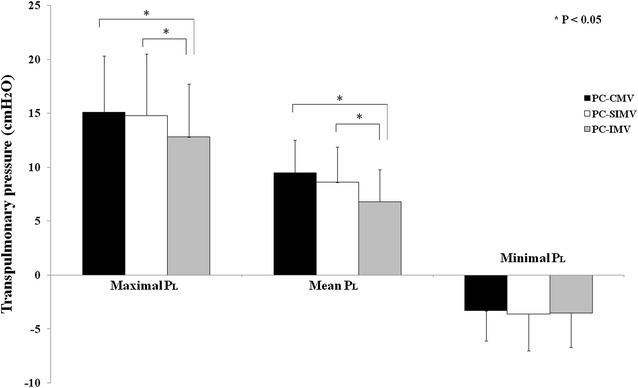
Maximal, mean, and minimum transpulmonary pressure (*P*_*L*_) during the three pressure-controlled modes of ventilation. *PC-CMV* pressure-controlled continuous mandatory ventilation, *PC-SIMV* pressure-controlled synchronized intermittent mandatory ventilation, *PC-IMV* pressure-controlled intermittent mandatory ventilation

The main results are shown in Table 2 and Figs. 2 and 3. The percentage of spontaneous breathing during PC-SIMV and PC-IMV was 6.8 and 17.4% of total minute ventilation. We found that average *V*_T_ and *V*_T_ per predicted body weight were significantly lower during PC-IMV in comparison with the two other modes (PC-IMV vs PC-CMV, *p* < 0.001; PC-IMV vs PC-SIMV, *p* = 0.049). Tidal volume variability was significantly higher during PC-IMV as compared with the other modes (PC-IMV vs PC-CMV, p = 0.001; PC-IMV vs PC-SIMV, *p* = 0.028) (Fig. 2). Total respiratory rate also significantly increased during PC-IMV in comparison with PC-SIMV and PC-CMV (PC-IMV vs PC-CMV, *p* = 0.007; PC-IMV vs PC-SIMV, *p* = 0.025).

**Table 2:** Breathing pattern, respiratory and hemodynamic variables during three pressure-controlled modes

	PC-CMV	PC-SIMV	PC-IMV
Tidal volume (mL)	482 ± 107	457 ± 133	387 ± 104*^,#^
Tidal volume per predicted body weight (mL/kg)	7.3 ± 1.4	7.0 ± 2.1	5.9 ± 1.5*^,#^
Tidal volume variability (%)	13.7 ± 13.7	21.6 ± 13.1	36.0 ± 18.0*^,#^
Maximal *P*_*L*_ (cmH_2_O)	15.5 ± 5.2	15.3 ± 5.7	13.3 ± 4.9*^,#^
Mean *P*_*L*_ (cmH_2_O)	9.8 ± 3.0	8.8 ± 3.3^γ^	7.0 ± 3.0*^,#^
Minimum *P*_*L*_ (cmH_2_O)	−3.2 ± 2.8	−3.5 ± 3.4	−3.5 ± 3.2
Δ*P*_*L*_ (cmH_2_O)	12.0 ± 6.9	11.9 ± 7.0	10.3 ± 4.6
Total respiratory rate (breaths/min)	22 ± 4	23 ± 6	27 ± 7*^,#^
Minute ventilation (L/min)	10.2 ± 2.1	9.8 ± 1.9	9.9 ± 2.0
PaO_2_/FiO_2_ ratio	216 ± 60	223 ± 55	218 ± 63
PaCO_2_ (mmHg)	48 ± 10	49 ± 11	50 ± 10
Arterial pH	7.37 ± 0.06	7.37 ± 0.07	7.36 ± 0.07
Mean arterial pressure (mmHg)	80 ± 10	80 ± 11	85 ± 14
Heart rate (beats/min)	96 ± 14	95 ± 13	96 ± 15

*PC*-*CMV* pressure-controlled continuous mandatory ventilation, *PC*-*SIMV* pressure-controlled synchronized intermittent mandatory ventilation, *PC*-*IMV* pressure-controlled intermittent mandatory ventilation

* *p* < 0.05, PC-CMV versus PC-IMV; ^#^ *p* < 0.05, PC-SIMV versus PC-IMV; ^γ^ *p* < 0.05, PC-CMV versus PC-SIMV

Average values of maximal *P*_*L*_ and the mean *P*_*L*_ during PC-IMV were significantly lower when compared to PC-CMV (*p* = 0.006) and PC-SIMV (*p* = 0.004), but no difference in minimum *P*_*L*_ during each mode of ventilation was found (Fig. 3). There was a nonsignificant trend toward a decreased Δ*P*_*L*_ at the end of inspiration with decreasing degree of inspiratory synchronization (PC-IMV vs PC-CMV, *p* = 0.144; PC-IMV vs PC-SIMV, *p* = 0.152). No difference in minute ventilation, PaO_2_/FiO_2_, PaCO_2_, and arterial pH was found between modes. In addition, no significant differences in mean arterial pressure and heart rate were found between the three PC modes (Table 2).

### Effect on patient’s inspiratory effort

Patient’s inspiratory effort determined by PTP_es_ was higher during PC-IMV in comparison with the two other modes (PC-IMV vs PC-CMV, *p* = 0.005; PC-IMV vs PC-SIMV, *p* = 0.023), as shown in Table 3. Compared to the two other modes, PTP_es_ increased by more than 15% in 10 patients and by more than 50% in 5 patients.

**Table 3:** Patient inspiratory effort [esophageal pressure–time product (PTP_es_)] and respiratory drive [airway occlusion pressure at 0.1 s (*P*_0.1_)] during three pressure-controlled modes

	PC-CMV	PC-SIMV	PC-IMV
PTP_es_ (cmH_2_O × s × min^−1^)	130 ± 101	150 ± 102	215 ± 154*^,#^
*P*_0.1_ (cmH_2_O)	2.6 ± 1.7	2.9 ± 1.9	3.7 ± 2.3*

*PC*-*CMV* pressure-controlled continuous mandatory ventilation, *PC*-*SIMV* pressure-controlled synchronized intermittent mandatory ventilation, *PC*-*IMV* pressure-controlled intermittent mandatory ventilation

* *p* < 0.05, PC-CMV versus PC-IMV; ^#^ *p* < 0.05, PC-SIMV versus PC-IMV

We found that *P*_0.1_, measured during manual occlusions, significantly increased from 2.6 ± 1.7 cmH_2_O during PC-CMV to 3.7 ± 2.3 cmH_2_O during PC-IMV (*p* = 0.048) (Additional file 1: Fig. S3). We observed a strong correlation between *P*_0.1_ and PTP_es_ with a correlation coefficient of 0.754 (*p* < 0.001). In addition, the area under the ROC curve for *P*_0.1_ to predict excess patient’s inspiratory effort was 0.93 (95% confidence interval, 0.85–1.00) (Additional file 1: Fig. S4). A cutoff value for *P*_0.1_ above 3.5 cmH_2_O had a sensitivity of 92% and specificity of 89% in predicting PTP_es_ > 200 cmH_2_O × s × min^−1^.

## Discussion

We found that spontaneous efforts during different PC modes with identical ventilator settings have very different effects on *V*_T_ and *P*_*L*_. PC-IMV has no synchronization and provides less *V*_T_ and *P*_*L*_ and more *V*_T_ variability than either PC-CMV or PC-SIMV, which have full or partial synchronization. No differences in terms of gas exchange and hemodynamics were found between the modes in this short-term study. The non-synchronized mode was, however, often associated with higher levels of patients’ effort. Inspiratory effort was strongly correlated with *P*_0.1_. In this context, *P*_0.1_ might be used to detect excessive inspiratory effort.

Patients with acute respiratory failure, in particular ARDS, should be ventilated with a lung-protective strategy to reduce the risk of ventilator-induced lung injury (VILI) and to improve survival [15–17]. Using low *V*_T_ and optimum PEEP to minimize *P*_*L*_ can mitigate VILI [18]. Although neuromuscular blocking agents can be used initially, allowing spontaneous breathing can reduce VIDD [6, 7, 19], improves lung aeration and oxygenation [5, 20–22], and may attenuate VILI especially when the degree of lung injury is moderate [23, 24]. PC modes have been increasingly used, in particular, after 48 h of mechanical ventilation [1] because it provides a variable flow rate and may well respond to patient’s demand and reduce work of breathing [2]. However, when patients breathe spontaneously during PC modes, the patient’s inspiratory effort can increase *P*_*L*_ and *V*_T_ which has the potential to worsen lung injury [25, 26].

Our study shows that the level of inspiratory synchronization should be considered when using a PC mode and probably individualized. Richard et al. [9] demonstrated on a bench that *V*_T_ and *P*_*L*_ significantly increased when the degree of synchronization increased. These findings are confirmed by the present study in that non-synchronized mode lowers the average *V*_T_ and *P*_*L*_ in comparison with synchronized mode. Variation in *V*_T_ and *P*_*L*_ may occur because of different breath types during PC-IMV, and higher distending pressure may develop during some breath types such as type B breath (Additional file 1: Fig. S1). However, the average inspiratory time in our study was around 1 s and patients had little chances to breathe at high pressure level. Furthermore, we did not add pressure support during PC-SIMV and PC-IMV limiting the chance of higher *V*_T_ and *P*_*L*_. In addition, variable *V*_T_ during non-synchronized modes may mimic a more natural breathing pattern and higher variability has been associated with improved respiratory mechanics and outcomes [27–30]. Calzia et al. [31] compared PC-SIMV with PSV in 19 patients after coronary artery bypass grafting. The results showed that *V*_T_ was lower during PC-SIMV (called “biphasic CPAP”) than during PSV (which could be considered as fully synchronized PC mode). Gama de Abreu and colleagues [32] also compared PC-SIMV to PSV in 10 anesthetized pigs with acute lung injury. They found that average *V*_T_ was higher during PSV compared to PC-SIMV, with no differences in terms of gas exchange and hemodynamics. These findings are in line with the results of our study. In contrast, a study by Yoshida et al. [33] conducted in 18 patients with ARDS compared a non-synchronized mode with PSV set to deliver equal mean Paw. Authors showed that lung aeration and oxygenation improved during the non-synchronized mode and no differences in hemodynamics were found between modes. Our results suggest that a non-synchronized mode may be considered to be used as a transition mode between fully controlled ventilation and the resumption of spontaneous efforts in order to reduce the risk of VILI in patients with ARDS or at high risk of ARDS.

PC-IMV provided less *V*_T_ and *P*_*L*_ than the other modes, but patient inspiratory effort frequently increased either because of the lack of synchronization between the patient and the ventilator or, more likely in some patients, because insufficient setting of mechanical ventilation was provided, increasing the drive to breathe [34, 35]. Calzia et al. [31] also found that PTP_es_ increased during PC-SIMV in comparison with PSV. Appropriate titration of sedative/analgesic drugs and/or adaptation of the level of ventilation (i.e. using higher respiratory rate) may alleviate the patient’s high inspiratory drive. This strategy should be considered when using partially or non-synchronized modes. Other approaches for alleviating patient inspiratory effort such as using higher PEEP, extracorporeal carbon dioxide removal, or partial neuromuscular blockade [36] may need to be explored in the future. In our study, we did not modify the backup respiratory rate, which was probably insufficient during this mode in some patients. Strong spontaneous efforts may worsen lung injury and overstretch the dependent lung zones because of a pendelluft phenomenon, especially when severe lung injury is present [25, 37]. A study by Güldner et al. [38] demonstrated that spontaneous ventilation during APRV improved oxygenation and reduced lung stress and strain regardless of the level of spontaneous effort. This latter finding may be explained by lowering *V*_T_ and *P*_*L*_ with non-synchronized mode. Spontaneous breathing during non-synchronized mode is recommended to be in the range of 10–30% of total minute ventilation to improve ventilation/perfusion matching and gas exchange and to avoid excessive work of breathing [39, 40]. In our study 16.7% of spontaneous breathing during PC-IMV is consistent with this suggestion to keep spontaneous breathing less than 30%. Thus, maintaining the advantages of non-synchronized modes while avoiding high respiratory effort merits to be attempted.

Of note, calculations of work of breathing using Campbell’s diagram and PTP_es_ are the gold standard for evaluating patient’s inspiratory effort but these techniques are not available at the bedside. *P*_0.1_ is a simple and noninvasive method, available on most modern ventilators, which evaluates the respiratory center drive [41]. Our study showed a good correlation between *P*_0.1_ and PTP_es_, confirming the results of previous studies conducted in different populations and with various ventilator modes [42–44]. We need to confirm that *P*_0.1_ can be a good surrogate marker of patient’s excessive inspiratory effort but it shows promising results to be used by clinicians to indicate when excessive levels of effort occur.

Our study is a short-term physiologic study and clinical outcomes were not evaluated, which limit the clinical conclusions that can be inferred from the study. The APRV mode used in this study was set to mimic the conventional ventilator setting. We did not measure respiratory mechanics to avoid sedation that may affect spontaneous breathing. We also did not measure biomarkers to assess the effect of inspiratory synchronization on lung injury. We need investigation in larger clinical studies and for longer periods of time to evaluate the impact of different types of PC mode, but we believe these data are useful to better understand how these modes can be used.

## Conclusions

Non-synchronized PC ventilation provides less *V*_T_, lower *P*_L_ and more breath to breath variability than partially and fully synchronized modes, despite identical ventilator settings. In this regard, this mode may help to protect the lungs and its use as a transition mode, between fully controlled ventilation and the resumption of spontaneous efforts. The risk is to increase patient’s effort, and therefore, a close monitoring of respiratory drive as well as acid–base and ventilation status is needed.

## Additional file

**Additional file 1.** Effect of inspiratory synchronization during pressure-controlled ventilation on lung distention and inspiratory effort.

## Abbreviations

ARDS: acute respiratory distress syndrome
APRV: airway pressure release ventilation
*P*_aw_: airway pressure
PC: pressure-controlled
PC-CMV: PC continuous mandatory ventilation
PC-IMV: PC intermittent mandatory ventilation
PC-SIMV: PC synchronized intermittent mandatory ventilation
PEEP: positive end-expiratory pressure
*P*_es_: esophageal pressure
*P*_*L*_: transpulmonary pressure
PSV: pressure support ventilation
PTP_es_: esophageal pressure–time product
*P*_0.1_: airway occlusion pressure at 0.1 s
ROC: receiver operating characteristic
VIDD: ventilator-induced diaphragmatic dysfunction
VILI: ventilator-induced lung injury
*V*_T_: tidal volume

## References

[1] Esteban A, Frutos-Vivar F, Muriel A, Ferguson ND, Peñuelas O, Abraira V (2013). Evolution of mortality over time in patients receiving mechanical ventilation. Am J Respir Crit Care Med.

[2] Rittayamai N, Katsios CM, Beloncle F, Friedrich JO, Mancebo J, Brochard L (2015). Pressure-controlled vs volume-controlled ventilation in acute respiratory failure: a physiology-based narrative and systematic review. Chest.

[3] Levine S, Nguyen T, Taylor N, Friscia ME, Budak MT, Rothenberg P (2008). Rapid disuse atrophy of diaphragm fibers in mechanically ventilated humans. N Engl J Med.

[4] Jaber S, Petrof BJ, Jung B, Chanques G, Berthet J-P, Rabuel C (2011). Rapidly progressive diaphragmatic weakness and injury during mechanical ventilation in humans. Am J Respir Crit Care Med.

[5] Putensen C, Mutz NJ, Putensen-Himmer G, Zinserling J (1999). Spontaneous breathing during ventilatory support improves ventilation–perfusion distributions in patients with acute respiratory distress syndrome. Am J Respir Crit Care Med.

[6] Sassoon CSH, Zhu E, Caiozzo VJ (2004). Assist-control mechanical ventilation attenuates ventilator-induced diaphragmatic dysfunction. Am J Respir Crit Care Med.

[7] Futier E, Constantin J-M, Combaret L, Mosoni L, Roszyk L, Sapin V (2008). Pressure support ventilation attenuates ventilator-induced protein modifications in the diaphragm. Crit Care Lond Engl.

[8] Kallet RH (2011). Patient-ventilator interaction during acute lung injury, and the role of spontaneous breathing: part 2: airway pressure release ventilation. Respir Care.

[9] Richard JCM, Lyazidi A, Akoumianaki E, Mortaza S, Cordioli RL, Lefebvre JC (2013). Potentially harmful effects of inspiratory synchronization during pressure preset ventilation. Intensive Care Med.

[10] Brochard L, Martin GS, Blanch L, Pelosi P, Belda FJ, Jubran A (2012). Clinical review: respiratory monitoring in the ICU—a consensus of 16. Crit Care Lond Engl.

[11] Baydur A, Behrakis PK, Zin WA, Jaeger M, Milic-Emili J (1982). A simple method for assessing the validity of the esophageal balloon technique. Am Rev Respir Dis.

[12] Akoumianaki E, Maggiore SM, Valenza F, Bellani G, Jubran A, Loring SH (2014). The application of esophageal pressure measurement in patients with respiratory failure. Am J Respir Crit Care Med.

[13] Sassoon CS, Light RW, Lodia R, Sieck GC, Mahutte CK (1991). Pressure–time product during continuous positive airway pressure, pressure support ventilation, and T-piece during weaning from mechanical ventilation. Am Rev Respir Dis.

[14] Jubran A, Tobin MJ (1997). Pathophysiologic basis of acute respiratory distress in patients who fail a trial of weaning from mechanical ventilation. Am J Respir Crit Care Med.

[15] Brower RG, Matthay MA, Morris A, Schoenfeld D, Thompson BT, Acute Respiratory Distress Syndrome Network (2000). Ventilation with lower tidal volumes as compared with traditional tidal volumes for acute lung injury and the acute respiratory distress syndrome. N Engl J Med.

[16] Determann RM, Royakkers A, Wolthuis EK, Vlaar AP, Choi G, Paulus F (2010). Ventilation with lower tidal volumes as compared with conventional tidal volumes for patients without acute lung injury: a preventive randomized controlled trial. Crit Care Lond Engl.

[17] Serpa Neto A, Simonis FD, Barbas CSV, Biehl M, Determann RM, Elmer J (2014). Association between tidal volume size, duration of ventilation, and sedation needs in patients without acute respiratory distress syndrome: an individual patient data meta-analysis. Intensive Care Med.

[18] Samary CS, Santos RS, Santos CL, Felix NS, Bentes M, Barboza T (2015). Biological impact of transpulmonary driving pressure in experimental acute respiratory distress syndrome. Anesthesiology.

[19] Gayan-Ramirez G, Testelmans D, Maes K, Rácz GZ, Cadot P, Zádor E (2005). Intermittent spontaneous breathing protects the rat diaphragm from mechanical ventilation effects. Crit Care Med.

[20] Varelmann D, Muders T, Zinserling J, Guenther U, Magnusson A, Hedenstierna G (2008). Cardiorespiratory effects of spontaneous breathing in two different models of experimental lung injury: a randomized controlled trial. Crit Care Lond Engl.

[21] Wrigge H, Zinserling J, Neumann P, Defosse J, Magnusson A, Putensen C (2003). Spontaneous breathing improves lung aeration in oleic acid-induced lung injury. Anesthesiology.

[22] McMullen SM, Meade M, Rose L, Burns K, Mehta S, Doyle R (2012). Partial ventilatory support modalities in acute lung injury and acute respiratory distress syndrome—a systematic review. PLoS ONE.

[23] Xia J, Zhang H, Sun B, Yang R, He H, Zhan Q (2014). Spontaneous breathing with biphasic positive airway pressure attenuates lung injury in hydrochloric acid-induced acute respiratory distress syndrome. Anesthesiology.

[24] Xia J, Sun B, He H, Zhang H, Wang C, Zhan Q (2011). Effect of spontaneous breathing on ventilator-induced lung injury in mechanically ventilated healthy rabbits: a randomized, controlled, experimental study. Crit Care Lond Engl.

[25] Yoshida T, Uchiyama A, Matsuura N, Mashimo T, Fujino Y (2012). Spontaneous breathing during lung-protective ventilation in an experimental acute lung injury model: high transpulmonary pressure associated with strong spontaneous breathing effort may worsen lung injury. Crit Care Med.

[26] Yoshida T, Uchiyama A, Matsuura N, Mashimo T, Fujino Y (2013). The comparison of spontaneous breathing and muscle paralysis in two different severities of experimental lung injury. Crit Care Med.

[27] Ma B, Suki B, Bates JHT (2011). Effects of recruitment/derecruitment dynamics on the efficacy of variable ventilation. J Appl Physiol Bethesda Md 1985.

[28] Lefevre GR, Kowalski SE, Girling LG, Thiessen DB, Mutch WA (1996). Improved arterial oxygenation after oleic acid lung injury in the pig using a computer-controlled mechanical ventilator. Am J Respir Crit Care Med.

[29] Suki B, Alencar AM, Sujeer MK, Lutchen KR, Collins JJ, Andrade JS (1998). Life-support system benefits from noise. Nature.

[30] Kiss T, Silva PL, Huhle R, Moraes L, Santos RS, Felix NS (2016). Comparison of different degrees of variability in tidal volume to prevent deterioration of respiratory system elastance in experimental acute lung inflammation. Br J Anaesth.

[31] Calzia E, Lindner KH, Witt S, Schirmer U, Lange H, Stenz R (1994). Pressure–time product and work of breathing during biphasic continuous positive airway pressure and assisted spontaneous breathing. Am J Respir Crit Care Med.

[32] de Abreu MD, Cuevas M, Spieth PM, Carvalho AR, Hietschold V, Stroszczynski C (2010). Regional lung aeration and ventilation during pressure support and biphasic positive airway pressure ventilation in experimental lung injury. Crit Care Lond Engl.

[33] Yoshida T, Rinka H, Kaji A, Yoshimoto A, Arimoto H, Miyaichi T (2009). The impact of spontaneous ventilation on distribution of lung aeration in patients with acute respiratory distress syndrome: airway pressure release ventilation versus pressure support ventilation. Anesth Analg.

[34] Marini JJ, Smith TC, Lamb VJ (1988). External work output and force generation during synchronized intermittent mechanical ventilation. Effect of machine assistance on breathing effort. Am Rev Respir Dis.

[35] Viale JP, Duperret S, Mahul P, Delafosse B, Delpuech C, Weismann D (1998). Time course evolution of ventilatory responses to inspiratory unloading in patients. Am J Respir Crit Care Med.

[36] Doorduin J, Nollet JL, Roesthuis LH, van Hees HWH, Brochard LJ, Sinderby CA (2017). Partial neuromuscular blockade during partial ventilatory support in sedated patients with high tidal volumes. Am J Respir Crit Care Med.

[37] Yoshida T, Torsani V, Gomes S, De Santis RR, Beraldo MA, Costa ELV (2013). Spontaneous effort causes occult pendelluft during mechanical ventilation. Am J Respir Crit Care Med.

[38] Güldner A, Braune A, Carvalho N, Beda A, Zeidler S, Wiedemann B (2014). Higher levels of spontaneous breathing induce lung recruitment and reduce global stress/strain in experimental lung injury. Anesthesiology.

[39] Carvalho NC, Güldner A, Beda A, Rentzsch I, Uhlig C, Dittrich S (2014). Higher levels of spontaneous breathing reduce lung injury in experimental moderate acute respiratory distress syndrome. Crit Care Med.

[40] Putensen C, Zech S, Wrigge H, Zinserling J, Stüber F, Von Spiegel T (2001). Long-term effects of spontaneous breathing during ventilatory support in patients with acute lung injury. Am J Respir Crit Care Med.

[41] Conti G, Antonelli M, Arzano S, Gasparetto A (1997). Measurement of occlusion pressures in critically ill patients. Crit Care Lond Engl.

[42] Mancebo J, Albaladejo P, Touchard D, Bak E, Subirana M, Lemaire F (2000). Airway occlusion pressure to titrate positive end-expiratory pressure in patients with dynamic hyperinflation. Anesthesiology.

[43] Alberti A, Gallo F, Fongaro A, Valenti S, Rossi A (1995). P0.1 is a useful parameter in setting the level of pressure support ventilation. Intensive Care Med.

[44] Berger KI, Sorkin IB, Norman RG, Rapoport DM, Goldring RM (1996). Mechanism of relief of tachypnea during pressure support ventilation. Chest.

