# Supplementary material for: Effect of inspiratory synchronization during pressure-controlled ventilation on lung distension and inspiratory effort
Source: Ann Intensive Care. 2017 Oct 6;7:100. doi: 10.1186/s13613-017-0324-z (PMC5630544; doi:10.1186/s13613-017-0324-z)
Supplement: Supplementary file 1 — Additional file 1. Effect of inspiratory synchronization during pressure-controlled ventilation on lung distention and inspiratory effort. [file 13613_2017_324_MOESM1_ESM.docx]

**Effect of inspiratory synchronization during pressure-controlled ventilation on lung distension and inspiratory effort**

**Authors**

Nuttapol Rittayamai, MD^1,2,3^, François Beloncle, MD^1,2,4^, Ewan C. Goligher, MD, PhD^1,5,6,7^, Lu Chen, MD^1,2^, Jordi Mancebo, MD, PhD^8,9^, Jean-Christophe M. Richard, MD, PhD^10,11^, Laurent Brochard, MD^1,2^

**METHODS**

**Calculation of esophageal pressure-time product (PTP_es_)**

Due to different patterns during partially and non-synchronized modes, with breaths that can occur at anytime with regards the two levels of ventilator pressure (mandatory breaths), we could not use the flow criterion to determine the beginning nor the end of the breath. We thus determined the respiratory cycle to calculate PTP_es_ as follows. The start of the inspiratory effort was determined at the instant of the P_es_ decay and the end of inspiration was determined to happen at the point of P_es_ that elapsed 25% of time from its maximum deflection to return to baseline (Fig.S2). This assumes that the final part of the esophageal curve is simply due to chest wall relaxation, which, however, could deserve further validation.

**Table S1** Nomenclature of pressure controlled mode of ventilation with different inspiratory synchronization on each ventilator brand. PC-CMV: pressure control continuous mandatory ventilation, PC-SIMV: pressure control synchronized intermittent mandatory ventilation, PC-IMV: pressure control intermittent mandatory ventilation.

| **Mode** | **Abbreviation** | **Manufacturer** |
| --- | --- | --- |
| **PC-CMV** | Pressure A/C | AVEA |
|  | PCV (assist control:ON) | Engstrom |
|  | BIPAPassist/PCV+assist/PC-AC | Evita-XL/Evita V500 |
|  | P-CMV | G5 |
|  | Pressure A/C | PB840 |
|  | PC | Servo-i |
| **PC-SIMV** | BiPhasic | AVEA |
|  | Bilevel | Engstrom |
|  | BIPAP/PCV+/PC-SIMV+ | Evita-XL/EVITA V500 |
|  | DuoPAP | G5 |
|  | BiLevel | PB840 |
|  | Bi-Vent | Servo-i |
| **PC-IMV** | APRV | AVEA |
|  | PCV (assist control:OFF)  APRV | Engstrom  EVITA-XL/EVITA V500 |
|  | APRV | G5 |
|  | - | PB840 |
|  | - | Servo-i |

**Fig.S1** Breath types during pressure control intermittent mandatory ventilation. Type A: spontaneous breathing during low pressure; type B: spontaneous breathing during high pressure; type C: breathing during transition from low to high pressure; type D: mandatory breath; type E: breathing during transition from high to low pressure; type E+A and A+B; hybrid types during two pressure levels


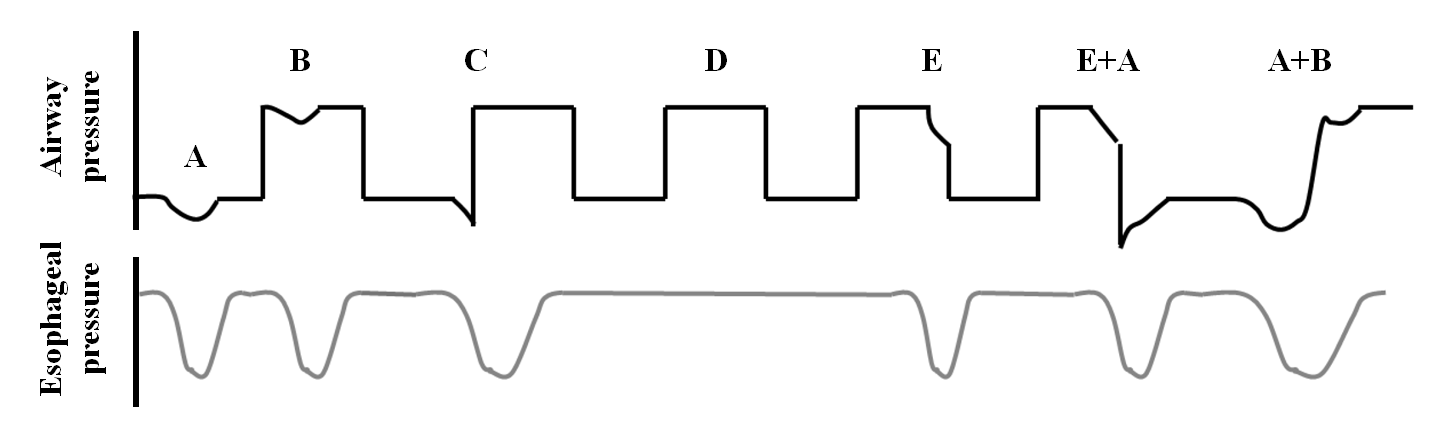


**Fig.S2** Calculation of esophageal pressure-time product during triggered breath and spontaneous breath. The beginning of the inspiratory cycle is the instant of the P_es_ decay (solid vertical line) and the end of the inspiratory cycle is the point of P_es_ that elapsed 25% of time from its maximum esophageal pressure deflection to return to baseline (dashed vertical line).


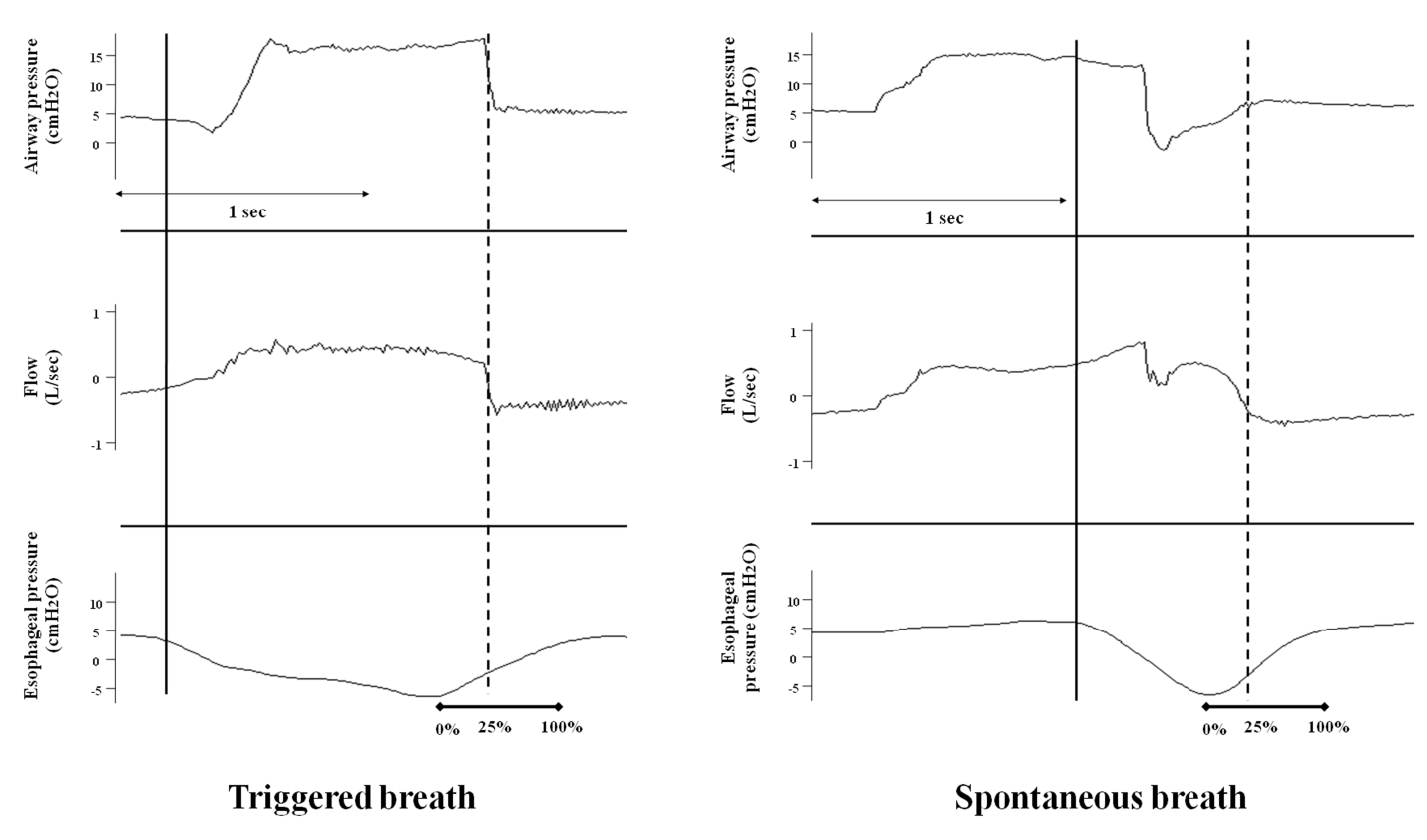


**Fig.S3** Airway occlusion pressure at 0.1 sec (P_0.1_) between fully, partially, and non inspiratory synchronized pressure controlled modes. PC-CMV: pressure control continuous mandatory ventilation, PC-SIMV: pressure control synchronized intermittent mandatory ventilation, PC-IMV: pressure control intermittent mandatory ventilation


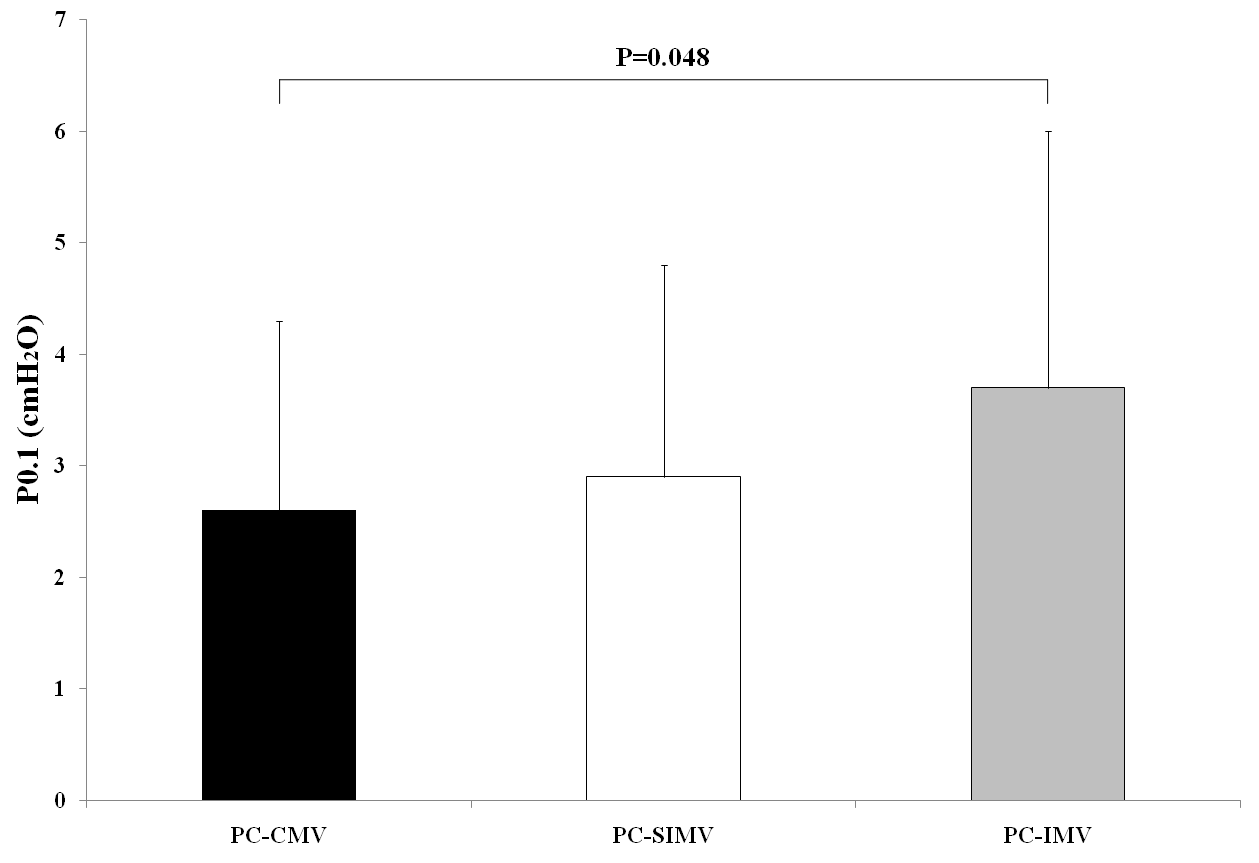


**Fig.S4** Area under receiver operating characteristic curves (AUC) for P_0.1_in predicting excess patient's inspiratory effort (PTP_es_> 200 cmH_2_O×sec×min^-1^).


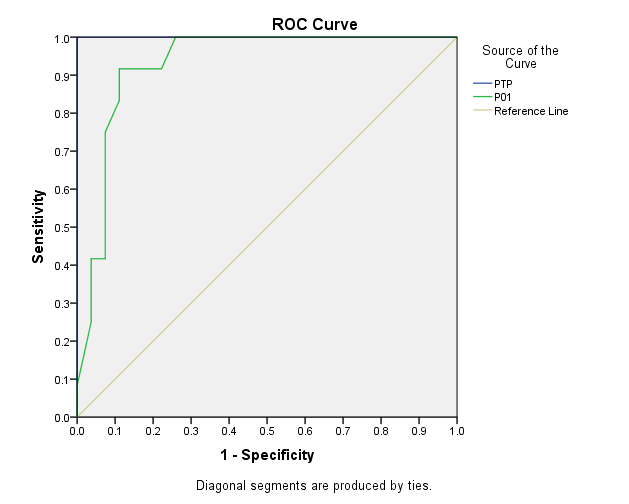


**AUC = 0.93 (95% CI, 0.84-1.00)**
